# Unveiling the molecular basis of lobeline's allosteric regulation of NMDAR: insights from molecular modeling

**DOI:** 10.1038/s41598-023-49835-2

**Published:** 2023-12-16

**Authors:** Chandran Remya, E. J. Variyar, R. V. Omkumar, C. Sadasivan, K. V. Dileep

**Affiliations:** 1 Laboratory for Computational and Structural Biology, Jubilee Centre for Medical Research, Jubilee Mission Medical College and Research Institute, Thrissur, Kerala 680005 India; 2 Department of Biotechnology and Microbiology, Kannur University, Dr. Janaki Ammal Campus, Thalassery, Kerala 670661 India; 3 Inter University Centre for Bioscience, Kannur University, Dr. Janaki Ammal Campus, Thalassery, Kerala 670661 India; 4 Neurobiology Division, Rajiv Gandhi Centre for Biotechnology, Thycaud PO, Trivandrum, Kerala 695014 India

**Keywords:** Computational biology and bioinformatics, Drug discovery, Structural biology

## Abstract

Neurological and psychiatric disorders contribute significantly to the global disease burden, adversely affecting the quality of life for both patients and their families. Impaired glutamatergic signaling is considered to be a major cause for most of the neurological and psychiatric disorders. Glutamate receptors are over activated in excitotoxic conditions, leading to dysregulation of Ca^2+^ homeostasis, triggering the production of free radicals and oxidative stress, mitochondrial dysfunction and eventually cell death. Excitotoxicity primarily results from the overactivity of NMDARs, a subtype of ionotropic glutamate receptors, due to their pronounced Ca^2+^ permeability and conductance characteristics. NMDAR antagonists are suggested to have therapeutic use as they can prevent excitotoxicity. Our previous studies demonstrated lobeline, an alkaloid, exerts neuroprotective action in excitotoxic conditions by blocking NMDAR. However, the atomic level interactions of lobeline with NMDAR was not characterized yet. Structural comparison of lobeline with a known NMDAR antagonist ifenprodil, followed by molecular docking and dynamics simulations revealed that lobeline could bind to the ifenprodil binding site i.e., in the heterodimer interface of GluN1-GluN2B subunits and exert ifenprodil like activities. By in silico structure guided modifications on lobeline and subsequent free energy calculations, we propose putative NMDAR antagonists derived from lobeline.

## Introduction

Glutamatergic neurons in the main excitatory system of the brain play a pivotal role in many neurophysiological functions^1^. Under normal conditions, glutamate, produces an excitatory response and plays a key role in primary perception and cognition in the brain^2^. This response is generated when glutamate interacts with its receptors. However, the excessive activation of glutamate receptors can lead to neuronal dysfunction and excitotoxicity^3,4^. Excitotoxicity is a condition at the central nervous system (CNS) characterized by progressive neuronal death due to an increased release and/or decreased uptake of excitatory amino acid transmitters, primarily glutamate^5^. The excitatory effects of glutamate are exerted via the activation of ionotropic receptors and metabotropic receptors linked to G-proteins^6^. Rapid excitation by glutamate, in turn, solely involves action at ionotropic receptors that include *N*-methyl-d-aspartic acid (NMDA), α-amino-3-hydroxy-5-methylisoxazole-4-propionate (AMPA) and kainic acid (KA) receptors^1,7^. These ionotropic receptors are ligand-gated ion channels permeable to various cations. The NMDA receptor (NMDAR) plays a pivotal role in both the formation and functions of the nervous system, as well as in neurotoxicity. The transmission of neurotransmitters facilitated by NMDAR holds utmost significance in fundamental brain development and functionality^8,9^.

The NMDARs are calcium (Ca^2+^) favoring glutamate-gated ion channels that are expressed in the CNS and were initially attributed to the neuronal death, owing to their heightened Ca^2+^ permeability and conductance properties^10^. NMDAR exhibit obligatory hetero-tetrameric assemblies, usually composed of two glycine binding GluN1 subunits and two glutamate-binding GluN2A-D subunits^11,12^. Sustained activation of GluN1/GluN2B NMDARs leads to elevated intracellular Ca^2+^ influx and protease activities. These processes initiate a cascade of events that ultimately culminate in cell death, either through apoptosis or necrosis^13^. These downstream effects encompass several critical processes, including mitochondrial membrane depolarization, generation of reactive oxygen species, activation of caspases, and cell death^14–16^. Even though the precise mechanisms by which NMDAR overactivation induces excitotoxicity may vary among different disorders, a substantial body of evidence implicates NMDAR-mediated excitotoxicity as a common pathway underlying the pathogenesis of numerous neurodegenerative conditions. These conditions range from acute events like stroke and brain trauma to chronic neurodegenerative disorders such as Alzheimer’s disease, Parkinson’s disease, and Huntington’s disease^17–22^.

NMDAR subunits exhibit a modular domain structure, featuring amino-terminal domains (ATDs) and ligand-binding domains (LBDs) situated on the extracellular region of the membrane. Subunits also consist of a transmembrane domain (TMD) that defines the ion channel pore, and a carboxy terminal domain (CTD) located within the cytoplasm^11,12^. A distinctive characteristic feature of NMDAR is their channel activity is allosterically regulated by small molecules that bind to the ATD, exhibiting subtype-specific effects. Ifenprodil and related phenylethanolamine compounds have gained significant attention due to their selective inhibition of GluN1/GluN2B NMDAR^23–25^. These compounds have been the subject of extensive research for their potential applications in the treatment of various neurological disorders and diseases.

Previously, we demonstrated that lobeline, a plant-based alkaloid present in *Lobelia inflata* blocks NMDAR activity and protects neurons from glutamate mediated excitotoxicity^26^. Lobeline is known for its CNS directed anxiolytic, anti-depressant and neuroprotective activities^27–35^. Even though it has been demonstrated that lobeline blocks NMDAR activity, the atomic level interactions of lobeline on NMDAR has not yet been explored. Moreover, elucidating the binding mode of lobeline on NMDAR is crucial, as NMDAR comprises various domains, and the binding of small molecules to each of these domains can have a regulatory effect on NMDAR activity^45–47^. In the present work, we made an attempt to study the structural basis of lobeline’s interactions with NMDAR. The structural similarity of lobeline with ifenprodil provided a rationale to propose lobeline’s binding at the heterodimer inter-subunit interface located between the ATDs of GluN1 and GluN2B containing NMDAR. To confirm lobeline’s binding at the ATD, molecular docking and molecular dynamics simulation were performed. Further, structure guided modification on lobeline was performed to propose novel lobeline derivatives with increased affinity towards NMDAR.

## Materials and methods

### Assessment of shape and chemical similarity of lobeline with ifenprodil

Shape-based screening is frequently utilized to identify molecules that exhibit similarities in both shape and electrostatic properties to a known binder^36^. In order to evaluate the similarity between lobeline and ifenprodil, we conducted shape and chemical-similarity assisted assessments. To perform a similarity check, the structures of both ligands were downloaded from PubChem database and were prepared using the LigPrep module, Schrödinger (Schrödinger, LLC, New York, USA). Ionized and tautomeric states of the ligands were generated using the Epik program and energy minimization was performed using optimized potential for liquid simulations (OPLS3) force field^37,41^. The shape-based flexible ligand superposition was done with the help of Schrödinger’s flexible alignment option^36^. Additionally, the Tanimoto similarity between lobeline and ifenprodil was assessed using the Swiss Similarity program^38,39^.

### Computational designing of putative lobeline derivatives

To enhance the affinity of lobeline binding, we employed computational techniques to design novel lobeline analogs, utilizing the ChemSketch program. The analogs were created by introducing different functional groups, and their binding orientations were assessed through molecular docking simulations. Subsequently, the free energy of binding for these modified lobeline derivatives was calculated using the MM-GBSA method.

### Binding studies of lobeline and its derivatives with NMDAR

For docking studies with NMDAR, crystal structure of the amino terminal domain of the NMDAR subunits, GluN1 and GluN2B in complex with ifenprodil (PDB ID: 3QEL) was employed directly rather without any structural modifications, or modeling the missing residues. A thorough structural analysis revealed no missing regions within 5 Å proximity of the ifenprodil binding site. Therefore, we assumed that the presence of these gaps is unlikely to impact the binding of ligands. Additionally, due to its better resolution when compared to other structures of ifenprodil bound NMDAR^23^ (site where ifenprodil bound was designated as S1) we used it for our studies. Further to explore possible binding of lobeline on other binding sites of NMDAR, we employed molecular docking by utilizing the following structures, the crystal structures of S-(+)-ketamine bound GluN1a–GluN2B NMDARs (PDB ID: 7SAC) (representing channel blocker bound conformation), crystal structure of the NMDAR GluN1 ligand binding domain in complex with 1-thioxo-1,2-dihydro-[1,2,4]triazolo[4,3-a] quinoxalin-4(5H)-one (PDB ID: 4KFQ) (representing glycine antagonist bound conformation) and structure of GluN1b-GluN2B NMDAR in complex with GluN2B antagonist SDZ 220-040 (PDB ID: 6WHX) (representing glutamate antagonist bound conformation). The corresponding binding sites in all these structures were named as S2–S4, respectively. The details of all the NMDAR crystal structures used in the docking studies are given in Table 1. Each receptor structures were prepared using protein preparation wizard by adding polar hydrogen atoms, incorporating proper protonation states of the residues, optimizing hydrogen bond networks and minimizing energy using an OPLS-3 force field with a RMSD cut off of 0.30 Å. The binding site was clearly defined using a grid which is generated on each minimized target structure around the centroid of the bound ligands and was used for the docking studies^42^. A focused docking of these ligands (both lobeline and its derivatives) was performed on each of the defined grid area of the selected protein structures using extra precision (XP) method employed in the Schrödinger maestro program^43^. We specifically employed XP because it does extensive sampling and have more sophisticated scoring function with greater requirements for ligand receptor shape complementarity^43^. In XP docking, only the active compounds show certain poses that avoid penalties and get good scores. This happens by making sure the protein and the ligand connect well through hydrophobic contact and hydrogen bonding. The binding energy of each protein–ligand complex was then predicted using Prime MM-GBSA method.

**Table 1:** Overview of the NMDAR crystal structures used in the molecular docking study.

Sl No.	PDB ID	Method	Resolution (Å)	Ligand	Subunits	Binding site	Referred as
1	3QEL	X-ray	2.60	QEL	GluN1–GluN2B	Hetero dimer interface-ATD	S1
2	7SAC	EM	3.69	JC9	GluN1–GluN2B	Channel vestibule-TMD	S2
3	4KFQ	X-ray	2.20	KFQ	GluN1	Glycine binding site-LBD	S3
4	6WHX	EM	4.09	QGP	GluN2B	Glutamate binding site-LBD	S4

*EM* electron microscopy; *QEL* ifenprodil; *JC9* S-(+)-ketamine; *KFQ* 1-sulfanyl[1,2,4]triazolo[4,3-a]quinoxalin-4(5H)-one; *QGP* 2S)-2-amino-3-[2ʹ,4ʹ-dichloro-4-hydroxy-5-(phosphonomethyl)biphenyl-3-yl]propanoic acid.

### Molecular dynamics simulation

Molecular dynamics (MD) simulations were performed to evaluate the stability and flexibility of the docked ligands at the ATD of NMDAR. The energetically most favorable pose was obtained when lobeline docked at the heterodimer interface of GluN1 and GluN2B (S1), which was used for the subsequent MD simulations. All MD simulations were performed by GPU accelerated Desmond software^44^ with the ligands. Prior to MD simulations, the system was set up using the "System Builder" in Desmond. The protein–ligand complex was immersed into an orthorhombic box with dimensions of 10 × 10 × 10 Å containing TIP3P explicit solvent model. The systems were further neutralized by adding Na^+^ or Cl^−^. In addition to that, 0.15 M of NaCl was also added to the system to provide a physiological environment. The number of atoms in each simulation system is given in the Table S1. Using the OPLS-2005 force field, the MD simulation was performed for 500 ns with a recording interval of 500 ps. The NPT ensemble was employed with a temperature fixed at 300 K and pressure at 1.01 bar. The thermostat and barostat methods used in the studies were ‘Nose–Hoover chain’ and ‘Martyna–Tobias–Klein’ respectively. RESPA was used for the integration and the integration time step was set at 2fs. Default settings were used for all other parameters.

### Residue decomposition analysis and in silico site directed mutagenesis

Post dynamic free energy calculation of the complexes was done using MM-GBSA method to understand the time evolution of the binding energy. Total 100 snapshots were extracted from MD simulation trajectories for the energy calculation. Additionally, per residue energy decomposition for each snapshot was also calculated to quantitatively measure the energy contributions of each residue in binding of lobeline and ifenprodil. To identify amino acids that exhibit significant energy contributions in the ligand binding were mutated to alanine based on a notion that alanine mutants may contribute little to the binding free energy. We then computed the difference in binding free energy before and after the mutation to evaluate the effect of these amino acid substitutions.

## Results and discussions

### Lobeline binds to the heterodimer inter-subunit interface at the ATD of NMDAR similar to ifenprodil

**Figure 1:**
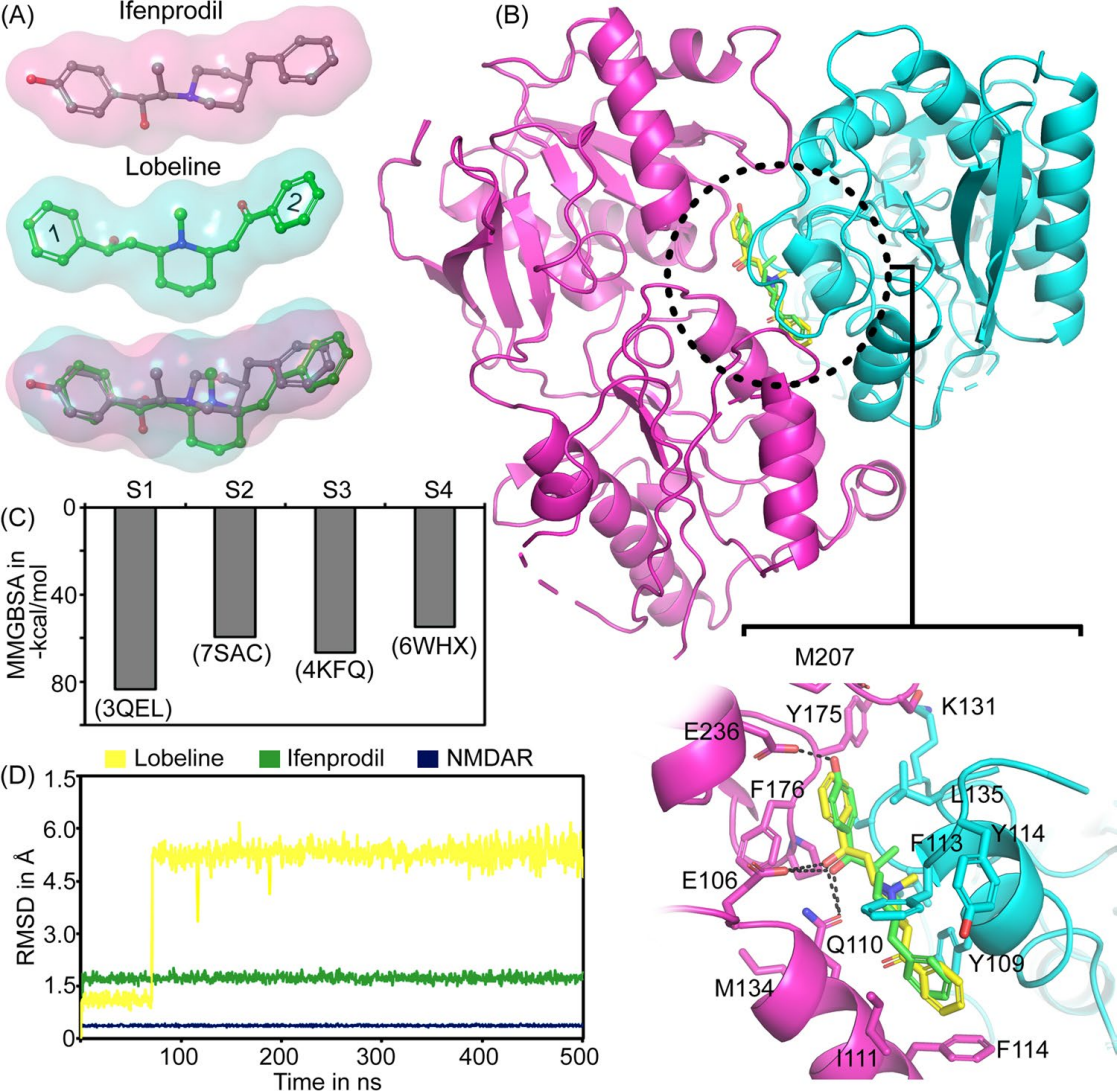
Binding of ifenprodil and lobeline on NMDAR. (**A**) The structure of ifenprodil, lobeline and their volume overlaps. Benzyl moieties in lobeline are marked as 1 and 2 and the structural modification was done on the ring 1. (**B**) The binding mode of ifenprodil (green) and lobeline (yellow) at the ATD of NMDAR. Compounds bind at the interface of GluN1 (represented in cyan) and GluN2B (represented in pink) subunits of ATD (S1). Hydrogen bonds are represented as black dashed lines. (**C**) Free energy of binding of lobeline at different binding sites of NMDAR. (**D**) The RMSD determined for ifenprodil and lobeline at the binding site (S1) of NMDAR during 500 ns simulation.

Our previous studies demonstrated lobeline’s neuroprotective activity against glutamate mediated excitotoxicity^26^. The cell-based studies provided a valid proof for the NMDAR channel blockade activity of lobeline^26^; however, the atomic level interaction of lobeline with NMDAR is unknown. Since lobeline was blocking the NMDAR channel activity, we hypothesized that lobeline may directly bind to the channel vestibule or bind to a site that can modulate the channel activity. It is noteworthy that the structure of lobeline (Fig. 1A) is very similar to a known antagonist of NMDAR called ifenprodil (Fig. 1A). The volume overlaps of these ifenprodil and lobeline (Fig. 1A), indicating a substantial level of shape similarity (87% similarity) between these ligands. Due to lobeline's significant structural similarity to ifenprodil, we speculated that lobeline could potentially bind to the same binding site on the NMDAR where ifenprodil binds. Ifenprodil binds to the heterodimer interface between the ATDs of GluN1 and GluN2B subunits (Fig. S1) and regulates the channel activity of NMDAR^23^. In order to determine the binding mode of lobeline towards the NMDAR, molecular docking study was carried in the S1, co-crystal structure of NMDAR-ifenprodil complex. The binding site was defined by assigning a grid on the crystal ligand, ifenprodil. The validity of docking protocol was confirmed by redocking ifenprodil with NMDAR. A comparison of the docked pose with the crystal pose did not show a noticeable RMSD between the ligands (Fig. S1), affirming the accuracy of the chosen docking protocol. The free energy of binding and the interaction pattern of ifenprodil and lobeline with NMDAR (Table 2 and Fig. 1B) suggested that lobeline shares a similar binding mode when compared to ifenprodil. The residues like Y109 from GluN1 and E236, F176, E106, F114 and Q110 of GluN2B subunit are majorly involved in ifenprodil binding.

**Table 2:** Comparative binding energetics of ifenprodil, lobeline, and its derivatives with NMDA Receptor.

Name	Glide score (Kcal/mol)	Evdw (Kcal/mol)	Ecoul (Kcal/mol)	Binding energy (∆G)(Kcal/mol)
Ifenprodil	− 13.05	− 38.50	− 21.12	− 91.99
Lobeline	− 10.07	− 42.33	− 10.02	− 83.50
Lob-1	− 12.82	− 44.25	− 17.27	− 95.10
Lob-2	− 11.42	− 44.24	− 17.27	− 94.68
Lob-3	− 07.82	− 46.80	− 11.89	− 86.12

*Evdw* van der Waals energy; *Ecoul* coulombic energy.

Both ligands possess similar interactions like stacking interactions with F114 and H-bond with Q110 (GluN2B). A cation–π interaction between positively charged piperidine ring nitrogen and Y109 (GluN1) was seen in the case of lobeline. On the other hand, a π-π interaction with F176 (GluN2B) and hydrogen bond (H-bond) between piperidinyl nitrogen and Q110 were observed in the case of ifenprodil. It is worth noting that the phenolic OH group of ifenprodil forms H-bond with E236 (GluN2B) which is missing in lobeline due to the absence of a hydroxyl group at the same position. This disparity in hydrogen bonding accounts for lower affinity of lobeline (glide score: − 10.07 kcal/mol) compared to ifenprodil (glide score: − 13.05 kcal/mol). This observation is also in accordance with the previous studies where lobeline exhibit lower potency (IC50: 75 μM)^26^ when compared to ifenprodil (IC50: 0.34 μM) in the in vitro conditions^23^.

Though lobeline has high structural similarity with ifenprodil, we also explored the binding potential of lobeline at other druggable sites in NMDAR (Fig. S2A). This includes channel vestibule of NMDAR (S2) and ligand binding domains of both GluN1 (S3) and GluN2B (S4). Our docking studies suggested that the binding of lobeline at other sites (Fig. S2B–D) are energetically weaker when compared to that in the S1, which further supported our claim that lobeline prefer to bind at the ifenprodil binding site i.e., at the S1 (Fig. 1C).

Further to understand the binding stability of lobeline in the S1, a 500 ns long MD simulation was performed. Crystal structure of the ATD of NMDAR in complex with ifenprodil was also simulated for 500 ns and used as a control experiment. While analyzing the RMSD of lobeline during the simulation, a significant structural deviation was observed (Fig. 1D). The RMSD of lobeline was found to stabilize around 1.2 Å for initial 65 ns and has undergone a large conformational rearrangement (average RMSD of 4.69 ± 1.48 Å), indicating a flexible landscape of lobeline at the binding site. In contrast, the binding of ifenprodil was stabilized below 2 Å (average RMSD of 1.72 ± 0.10 Å) throughout the simulation period attributed to the stable binding and higher affinity of ifenprodil than lobeline (Fig. 1D). The protein RMSD was below 0.5 Å during the entire period of simulation.

### Charged and polar residues play an important role in lobeline binding

**Figure 2:**
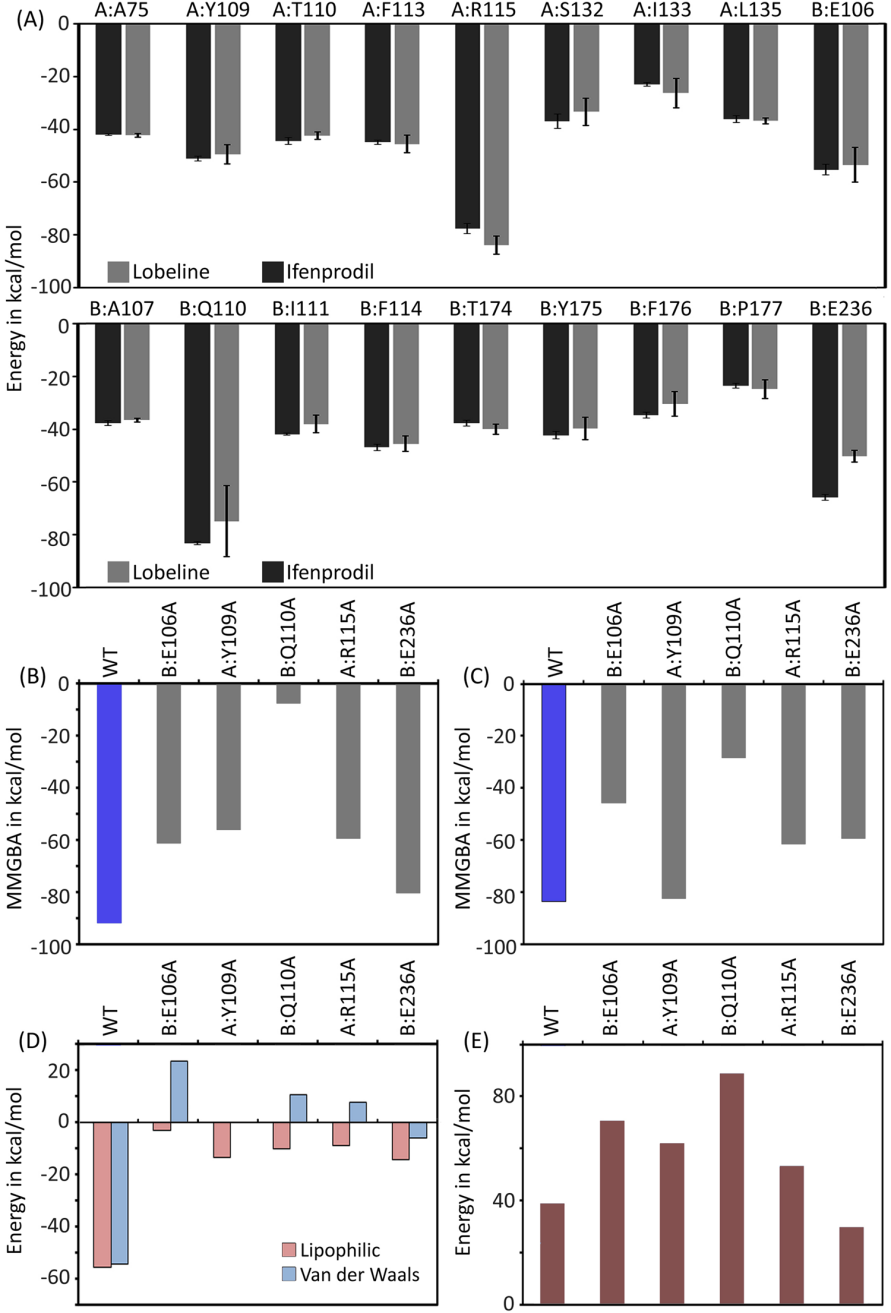
Residue level decomposition energy analysis. (**A**) The per residue energy decomposition analysis of interacting residues upon binding to ifenprodil (black bars) and lobeline (grey bars) with NMDAR at S1. Binding energies of lobeline (**B**) and ifenprodil (**C**) towards wildtype (WT) and five mutants (E106A, Y109A, Q110A, R115A and E236A) calculated using Prime MM-GBSA method. (**D**) The energy contributions from lipophilic and van der Waals contacts upon binding of lobeline with wild type and mutants. (**E**) The ligand strain energies calculated for lobeline in the wild type and mutant NMDAR complexes.

The per residue decomposition energy calculated from the MM-GBSA analysis of ifenprodil–NMDAR complex and lobeline–NMDAR complex is depicted in Fig. 2A. Five residues such as E106 (GluN2B), Y109 (GluN1), Q110 (GluN2B), R115 (GluN1) and E236 (GluN2B) that exhibit highest energy contributions towards the lobeline-NMDAR and ifenprodil-NMDAR complex formation were selected for in silico mutagenesis to further validate the significance of each residue in binding process. Five mutants such as E106A, Y109A, Q110A, R115A and E236A were prepared as described previously (See the section binding studies of lobeline and its derivatives with NMDAR). Further, the molecular docking of lobeline was done on S1, free energy of binding was deduced and compared with that of wild type. The binding energy of ifenprodil and lobeline towards mutants is presented in Fig. 2B,C, respectively. Lobeline interacts with Y109A mutant NMDAR with relatively similar strength as compared to that in wild type. However, the binding was significantly affected in the presence of other mutants such as Q110A, R115A, E106A and E236A mutants (Fig. 2C). The binding energy of lobeline is ~ 3-fold weaker in the case Q110A when compared to that in the wild type, indicating the crucial role of Q110 in determining the binding of lobeline. The disparity in binding energy highlights the significant role of charged and polar residues in lobeline binding. We noted a disruption of the H-bond interaction with Q110 in the Q110A mutant and the lack of cation-π interaction in the Y109A mutant. Furthermore, there was a notable differences in the lipophilic and van der Waals energy in the case of lobeline–mutant NMDAR complexes (Fig. 2D). Importantly, the ligand strain energy was consistently higher in all the mutants except E236A when compared to the binding of lobeline with the wild type (Fig. 2E). Typically, the strained conformations of the ligands results from adopting an unfavorable high-energy torsion angles during the binding process, leading to an increase in the strain energy of the ligands. The higher ligand strain energy in presence of the mutants indicates the significance of these residues in achieving the optimal binding poses.

### Structure guided modification on lobeline improved the binding stability at ATD of hetero dimer inter-subunit interface

In vitro and in silico studies showed that lobeline possesses a moderate antagonistic activity against NMDAR when compared to ifenprodil. The detailed analysis of the binding mode of lobeline with NMDAR pointed out that binding affinity of lobeline could be improved through structure-based drug design approach. The per residue binding energy decomposition analysis and in silico site-directed mutagenesis provided insights into the significance of four specific residues: E106, Q110, R115 and E236, in the binding of lobeline (Fig. 2A,B). The energy contributions of these residues were higher in the case of binding of lobeline and ifenprodil (Fig. 2A). We also observed that mutating these residues into alanine significantly affected the binding energies, suggesting the role of these residues in the binding of lobeline and ifenprodil (Fig. 2B).

**Figure 3:**
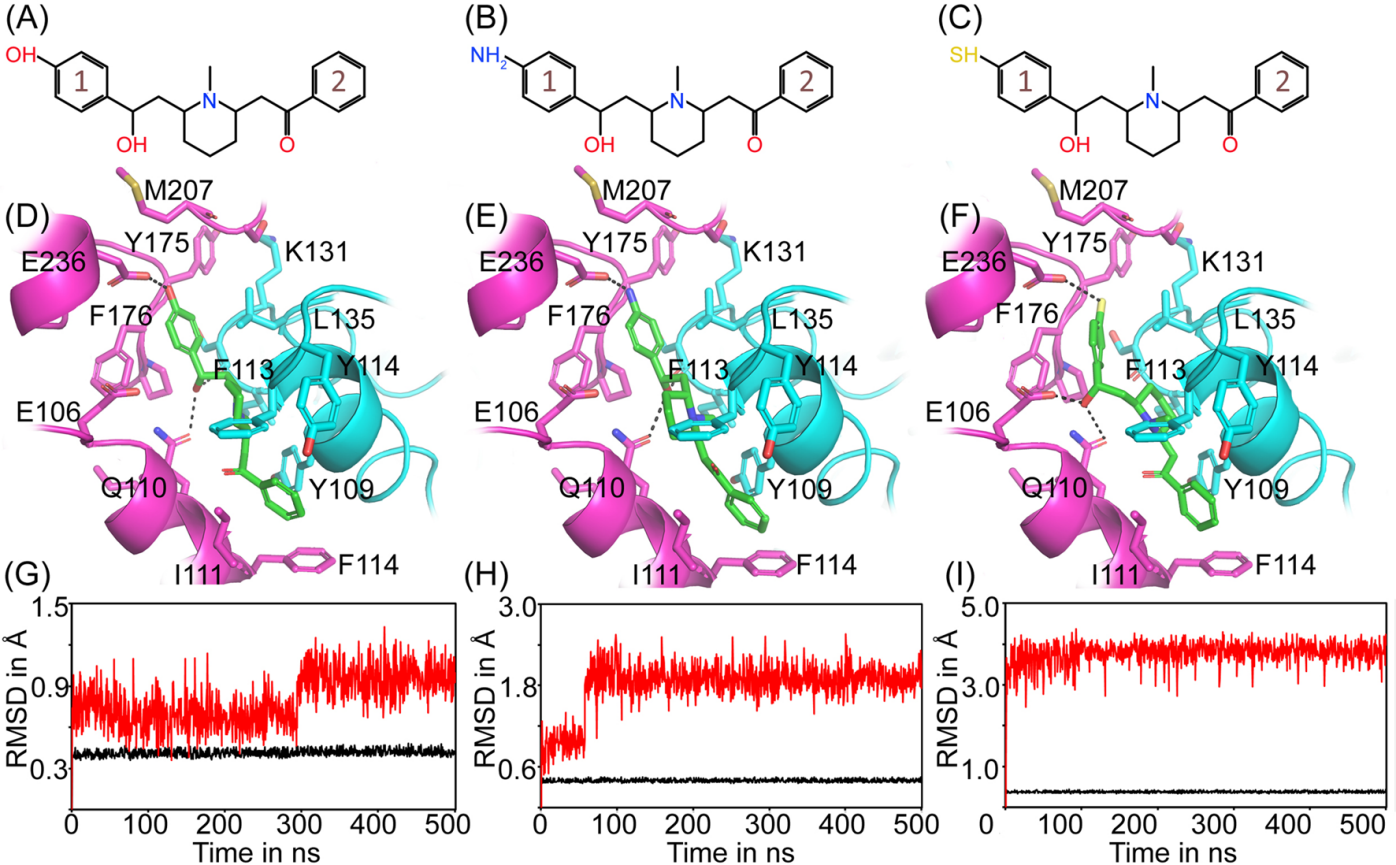
In silico based structural modifications on lobeline. The chemical structure of Lob-1 (**A**), Lob-2 (**B**) and Lob-3 (**C**). The benzyl moieties in each structure were marked as 1 and 2, the substitutions were done on the para position of benzene ring marked as 1. Binding mode of Lob-1 (**D**), Lob-2 (**E**) and Lob-3 (**F**) in the ATD interface of GluN1-GluN2B containing NMDAR. The ligands are shown in green stick model and protein is shown in cartoon representation. The cyan and pink color denotes GluN1 and GluN2B subunits respectively. The interacting residues are given in the stick model. Hydrogen bonds were represented as black dashed lines. The RMSD plot determined for Lob-1 (**G**), Lob-2 (**H**) and Lob-3 (**I**) at the binding site of NMDAR during 500 ns simulation. The black and red color represents the protein and ligand RMSD respectively.

Similar to ifenprodil, lobeline was also found to interact with E106 and Q110. The R115 primarily engages in van der Waals interactions with the ligands. However, due to the absence of a phenolic group in lobeline's structure, it is unable to make H-bond with E236 as observed in the ifenprodil binding (Fig. 1B). Prior mutagenesis studies have already demonstrated the critical role of E236 in ifenprodil-mediated inhibition^40^. Consequently, it is hypothesized that incorporating a functional group capable of forming a H-bond with E236 may potentially enhance the binding affinity of lobeline towards NMDAR. Thus, we incorporated a hydroxyl group and its isosteric groups on the corresponding position (i.e., on the para position of benzene ring marked as 1 on the lobeline skeleton, see Fig. 1A) and their interactions with NMDAR at the S1 (hetero dimer interface between GluN1 and GluN2B) were studied. The derivatives of lobeline were named as Lob-1 (having –OH group on the para position of benzene ring marked as 1), Lob-2 (having –NH2 group on the para position of benzene ring marked as 1) and Lob-3 (having –SH group on the para position of ring benzene marked as 1) are represented in Fig. 3A–C, respectively. The Glide docking descriptors generated are given in the Table 2 and the binding mode of Lob-1, Lob-2 and Lob-3 at the S1 is shown in Fig. 3D–F, respectively.

The stability of these derivatives at the binding site of NMDAR was further assessed using 500 ns MD simulations and their RMSD was plotted. The RMSD plot of Lob-1 (Fig. 3G), Lob-2 (Fig. 3H) and Lob-3 (Fig. 3I) suggested that the structural modification not only improved the binding affinity but also provided stability for the derivatives when compared to lobeline (Fig. 1D). During the simulation, Lob-3 displayed more fluctuations with an average RMSD of 3.77 ± 0.30 Å (Fig. 3I). Conversely, the orientation of Lob-2 remained relatively stable throughout the simulation, with an average RMSD of 1.78 ± 0.36 Å (Fig. 3H). Among the derivatives, Lob-1 exhibited relatively stable binding, with an average RMSD of 0.80 ± 0.18 Å (Fig. 3G). These findings were further supported by the ligand torsion profile, illustrating the increased dynamics of lobeline compared to its derivatives (Figs. S3–Figs. S7). Specifically, the phenyl ethanol moiety of lobeline demonstrated greater rotational freedom due to fewer interactions with the protein, while the remaining portion of the molecule experienced minimal fluctuations (Fig. S4).

### Intermolecular contacts were enhanced by structural modifications

**Figure 4:**
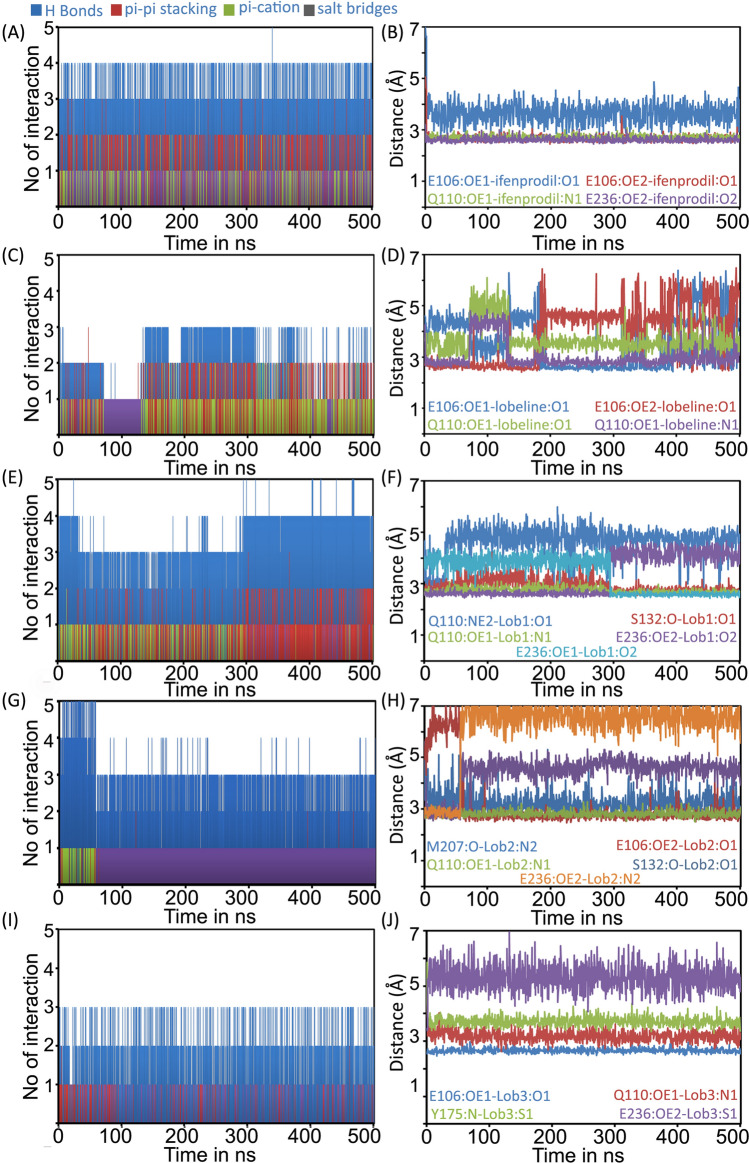
Time evolution of the inter-molecular contacts formed between the ligand and protein. No. of contacts formed between (**A**) ifenprodil, (**C**) Lobeline, (**E**) Lob-1, (**G**) Lob-2 and (**I**) Lob-3 with NMDAR during the simulation period. The dynamics of H-bond formation between the ifenprodil (**B**), Lobeline (**D**), Lob-1 (**F**), Lob-2 (**H**) and Lob-3 (**J**) with ATD of NMDAR.

We then critically analyzed the intermolecular interactions between lobeline and its derivatives at the hetero-dimer interface of NMDAR. Time evolution of interaction patterns suggests that the binding of all ligands are primarily characterized by H-bonds and stacking interactions (Fig. 4A,C,E,G,I). However, we observed a decrease in the number of contacts, especially H-bonds, in the case of lobeline (Fig. 4C), when compared to ifenprodil (Fig. 4A) and its derivatives (Fig. 4E,G,I). As mentioned earlier, there were noticeable fluctuations observed around 65 ns in the case of lobeline (Fig. 1D). This is clearly visible in the interaction plot, as most of the interactions were disrupted during this time period (Fig. 4C). During the simulation, lobeline formed two H-bonds, with Q110 and E106, which remained stable for approximately 82% and 79% of the simulation duration, respectively. To further assess the stability of these H-bond formations, we plotted the time evolution of H-bond distances between the ligand atoms and protein residues. Ifenprodil displayed stable H-bond formations with the interacting residues such as E106, Q110 and E236, as depicted in Fig. 4B. This observation aligns well with the torsion profile of ifenprodil (Fig. S3), indicating a stable conformation of ifenprodil in its binding orientation. The timeline depiction of H-bond distances involving E106 and Q110 with lobeline revealed that lobeline initially established two H-bonds with these residues (Fig. 4D). However, the H-bond between the piperidinyl nitrogen atom and OE1 of Q110 was disrupted (H-bond distance > 3.5 Å) between the 65–110 ns time frame. Later, lobeline re-established this H-bond for the rest of the simulation, indicating the flexible nature of lobeline within the binding site (Fig. S4). Additionally, instances of polar contacts between the hydroxyl group of lobeline and Q110 were also observed. Furthermore, the H-bond analysis revealed that the H-bond of the hydroxyl group of lobeline with E106 oscillated between its OE1 and OE2 atoms. The binding energy of lobeline, calculated from the simulation trajectories, was determined to be − 71.99 ± 10.01 kcal/mol.

Ifenprodil demonstrated stable H-bond formations with residues Q110 and E236 throughout the entire simulation period (Fig. 4B). In addition to the residue contacts observed in the crystallographic structures, we also observed H-bond with E106, which persisted in approximately 98% of the simulations. The H-bond formation predominantly involved the OE2 atom of E106, although instances of H-bonds with the OE1 atom of E106 were also observed (Fig. 4B). Furthermore, the stability of ifenprodil at the ATD was supported by a network of hydrophobic interactions with F176 (observed in 88% of the simulation time), F114 (observed in 45% of the simulation time), and Y109 (observed in 79% of the simulation time). These interactions contributed to the overall stability of ifenprodil in the binding pocket of the ATD (Fig. 1D). The binding energy of ifenprodil, calculated from the simulation trajectories is − 99.59 ± 7.52 kcal/mol.

Similar to ifenprodil, Lob-1 exhibited a comparable pattern of H-bond interactions, which correlated with the stability of its binding as inferred from the RMSD plot (Fig. 3G). The binding energy of Lob-1 (− 97.95 ± 13.17 kcal/mol) is very close to the binding energy of ifenprodil. As mentioned earlier, the Lob-1 formed stable H-bonds (> 90% of the simulation time) through its piperidinyl nitrogen, hydroxyl and phenyl moiety with residues Q110, S132, and E236 of NMDAR, resulting in a stronger binding (Fig. 4F). In addition to the H-bonds, Lob-1 is also engaged in π–π interactions with F176 and F114. These interactions also contributed to the binding stability, accounting for approximately 45% and 47% of the simulation time, respectively. Overall, these findings indicate that Lob-1 shares similarities with ifenprodil in terms of its stable H-bond interactions and the presence of important hydrophobic interactions (Fig. 4E). The conformational evolution of rotatable bonds displayed a relatively greater movement for the hydroxyl group in Lob-1 than its other counterparts (Fig. S5).

The binding of Lob-2 is characterized by H-bonds with E236, S132 and Q110 (Fig. 3E). However, the H-bond formation with E236 and S132 was observed only for initial ~ 60 ns and later the ligand underwent a conformational change that disrupted these H-bond formation (Fig. 4H). The stability of these H-bonds is influenced by the greater rotational motion experienced by the amine moiety and hydroxyl group, as depicted in Fig. S6. It is noteworthy that the ligand rearranged its position in such a way that the –OH group makes H-bond with E106 (persists in about 87%) and the amine group makes H-bond with M207 (persists in about 78%) respectively. The H-bond formation of piperidinyl nitrogen with Q110 was found to be stable during the simulation period. The binding is further supported by hydrophobic interactions with residues such as Y109, A107, I111, L135, F176 and F114 and salt bridge formation with E106 (Fig. 4G). The binding energy calculated is − 86.65 ± 13.30 kcal/mol.

The dynamics of Lob-3 shows that it undergoes a large conformational change from its docked pose (Fig. 3F) up to 4 Å and then equilibrium for the rest of the simulation time. We observed that the H-bond with E236 was abolished during the initial period of simulation due to this conformational change (Fig. 4J). However, the hydroxyl group makes a stable H-bond formation with E106. The binding is further supported by H-bond with Q110 (persists in about 46% of simulation time) and Y175 (persists in about 55% of simulation time) and π–π interaction with F176 and F114. The binding energy calculated is − 84.36.58 ± 9.44 kcal/mol. Overall the binding of Lob-3 is stabilized by H-bonds and stacking interactions (Fig. 4I). The exceptional stability of ligand torsion angles (Fig. S7) indicated that conformations of rotatable bonds in Lob-3 were stable while the ligand itself initially underwent a change in its binding orientation.

Taken together, the dynamics simulation studies indicated that the modifications improved binding stability and affinity of the ligands towards NMDAR. Notably, the H- bond interactions with E236, Q110 and E106 seems to be crucial for the binding stability of these ligands.

## Conclusions

This study investigated the binding potential of lobeline specifically at the heterodimer interface of the ATD, focusing on the GluN1 and GluN2B-containing NMDAR. Molecular docking studies suggested that lobeline adheres to the ATD in a manner similar to ifenprodil. However, it became apparent from our in silico studies that lobeline displayed a lower affinity and stability when compared to ifenprodil. This was underscored by higher RMSDs, primarily attributed to the absence of H-bond formation with E236, a characteristic interaction observed with ifenprodil. To enhance the binding stability and affinity of lobeline, structural modifications were introduced, including the addition of a hydroxyl group and its bioisosteres, resulting in the formation of an H-bond with E236. Analysis of RMSD plots and binding free energy profiles indicated that these newly designed derivatives exhibited improved binding stability and affinity when compared to lobeline. Analysis of ligand torsion profiles suggested a reduced conformational freedom for these new derivatives at the NMDAR binding site, contributed to the improved stability. Additionally, H-bond analysis highlighted the pivotal role of interactions with Q110 and E106 in the binding stability of the modified compounds. Although the in vitro studies have shown that lobeline can antagonize NMDAR activity, the structural elements that block the NMDAR activity was previously unknown. Thus, the current study that explains lobeline's atomic-level interactions with NMDAR provides a structural framework in the realm of future drug discovery, particularly in the context of diseases where excitotoxicity plays a pivotal role in the progression.

### Supplementary Information

Supplementary Information.

## Data Availability

The datasets used and/or analyzed during the current study available from the Corresponding author on reasonable request.
