# Supplementary material for: Unveiling the molecular basis of lobeline's allosteric regulation of NMDAR: insights from molecular modeling
Source: Sci Rep. 2023 Dec 16;13:22418. doi: 10.1038/s41598-023-49835-2 (PMC10725453; doi:10.1038/s41598-023-49835-2)
Supplement: Supplementary file 1 — Supplementary Information. [file 41598_2023_49835_MOESM1_ESM.pdf]

Unveiling the Molecular Basis of Lobeline's Allosteric Regulation of NMDAR: Insights from Molecular Modeling

Chandran Remya[1], E.J. Variyar[2], R.V. Omkumar[4], C. Sadasivan[2, 3] and K.V. Dileep[1*]

[1.] Laboratory for Computational and Structural Biology, Jubilee Centre for Medical Research, Jubilee Mission Medical College and Research Institute, Thrissur, Kerala, India. Pin 680005.

[2.] Department of Biotechnology and Microbiology, Kannur University, Dr. Janaki Ammal Campus, Thalassery, Kerala India. Pin 670661.

[3.] Inter University Centre for Bioscience, Kannur University, Dr. Janaki Ammal Campus, Thalassery, Kerala 670661, India.

[4] Neurobiology Division, Rajiv Gandhi Centre for Biotechnology, Thycaud PO, Trivandrum, Kerala 695014, India.

[*] For correspondence: Email: dileepkvijayan@gmail.com or dileepkvijayan@jmmc.ac.in

Key words: Lobeline, NMDA receptor, Excitotoxicity, neuroprotection, Molecular dynamics, Alzheimer’s disease, Ifenprodil

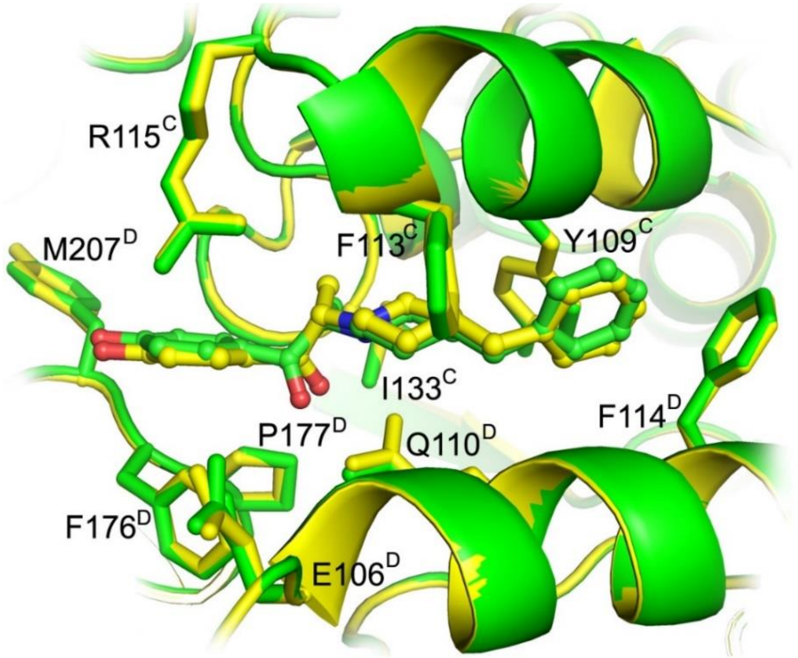

Figure S1: Overlay of ifenprodil (represented as ball and stick) conformation in both the docked state (green) and crystal (yellow) pose within the NMDAR. The NMDAR is represented in a cartoon model. Chain information is also mentioned as superscript where C and D indicates GluN1 and GluN2B respectively.

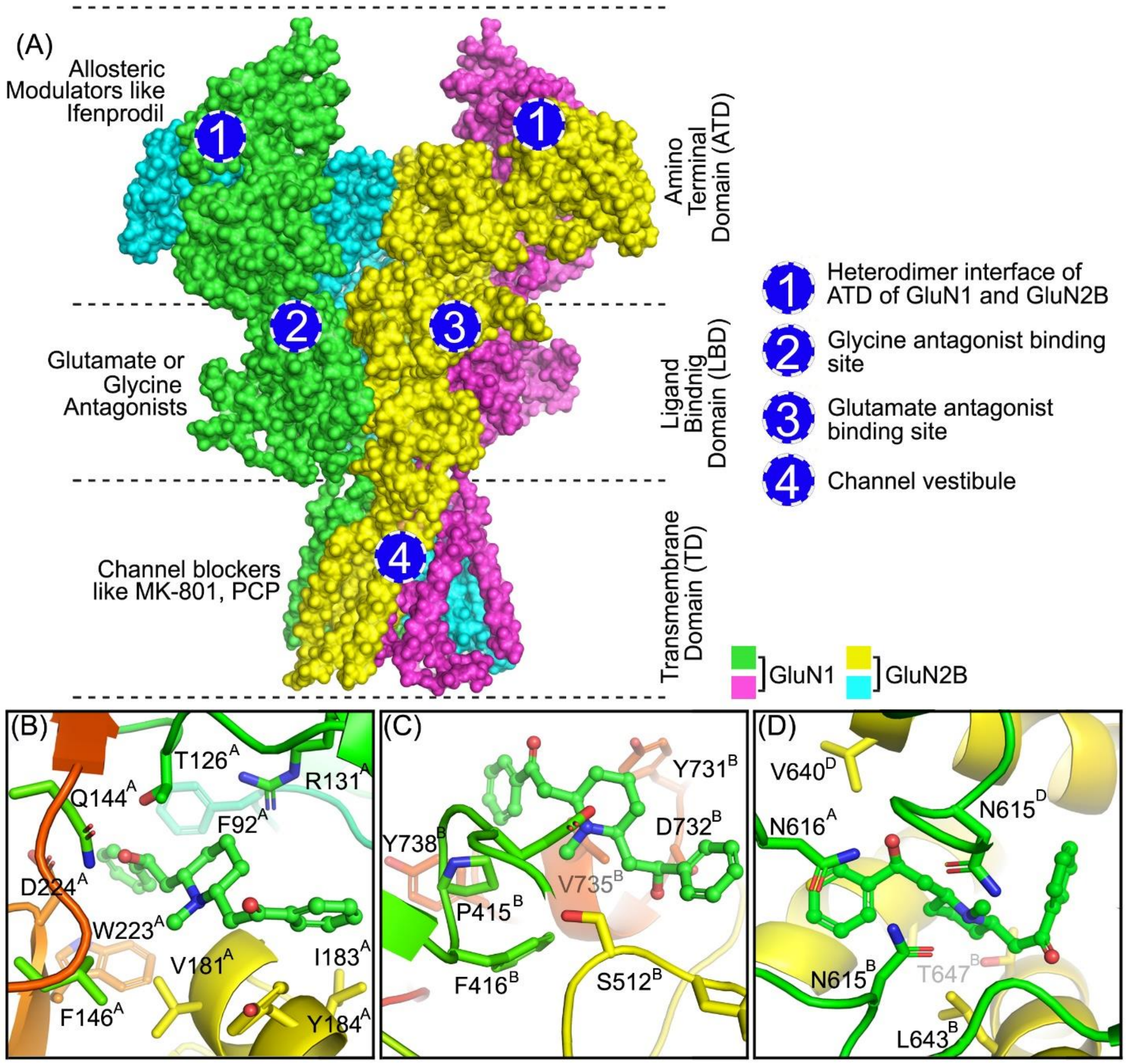

Figure S2: Three-dimensional structure of GluN1-GluN2B NMDAR subtype. Different pharmacological site representing the heterodimer interface of ATD of GluN1 and GluN2B, glycine antagonist and glutamate antagonist binding site and channel vestibule on NMDAR is marked (A). Binding of lobeline on glycine binding site (B), glutamate antagonist binding site (C) and at the channel vestibule (D) on NMDAR. Lobeline is depicted in ball and stick model, while the protein residues are shown in stick and cartoon model. Binding of lobeline at ATD is shown in Figure 1B. In Figure B-D, the chain information is given as superscripts, where A indicates GluN1; B and D indicates GluN2B.

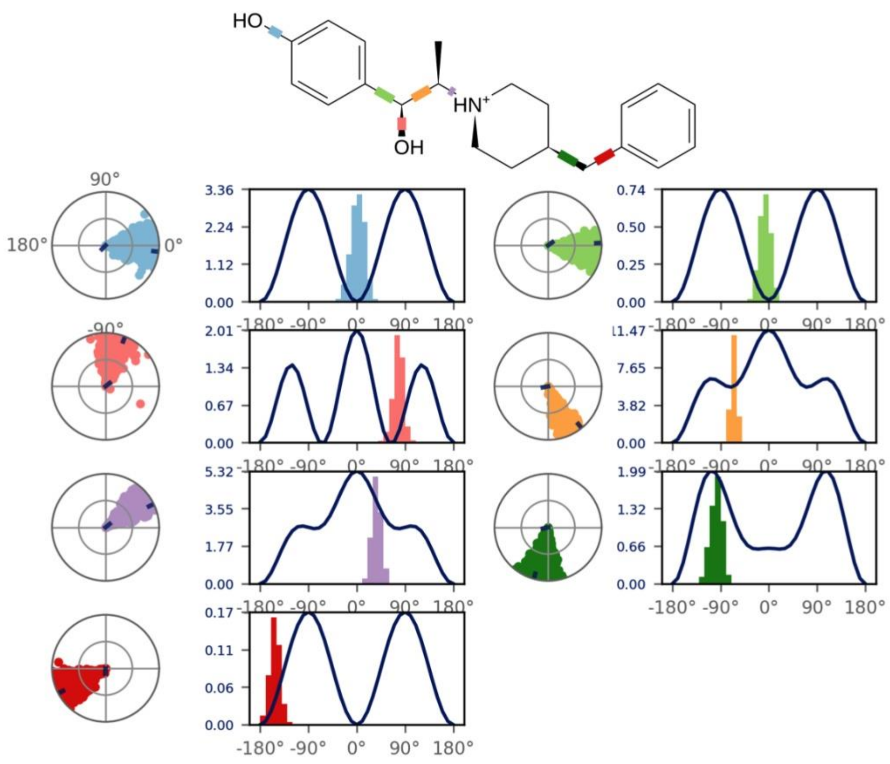

Figure S3: Torsion profile of the ifenprodil during the simulation. The torsion plot summarizes the conformational evolution of every rotatable bond (RB) of ifenprodil throughout the simulation trajectory. 2D structures of the ligands with color-coded rotatable bonds are shown. Radial plots describe the conformation of the torsion throughout the course of the simulation. Time evolution is plotted radially outwards from the center of the radial plot. The bar plots summarize the information presented in the dial plots by showing the probability density of the torsion. The values of the torsional potential are given in the Y-axis, and are expressed in kcal/mol.

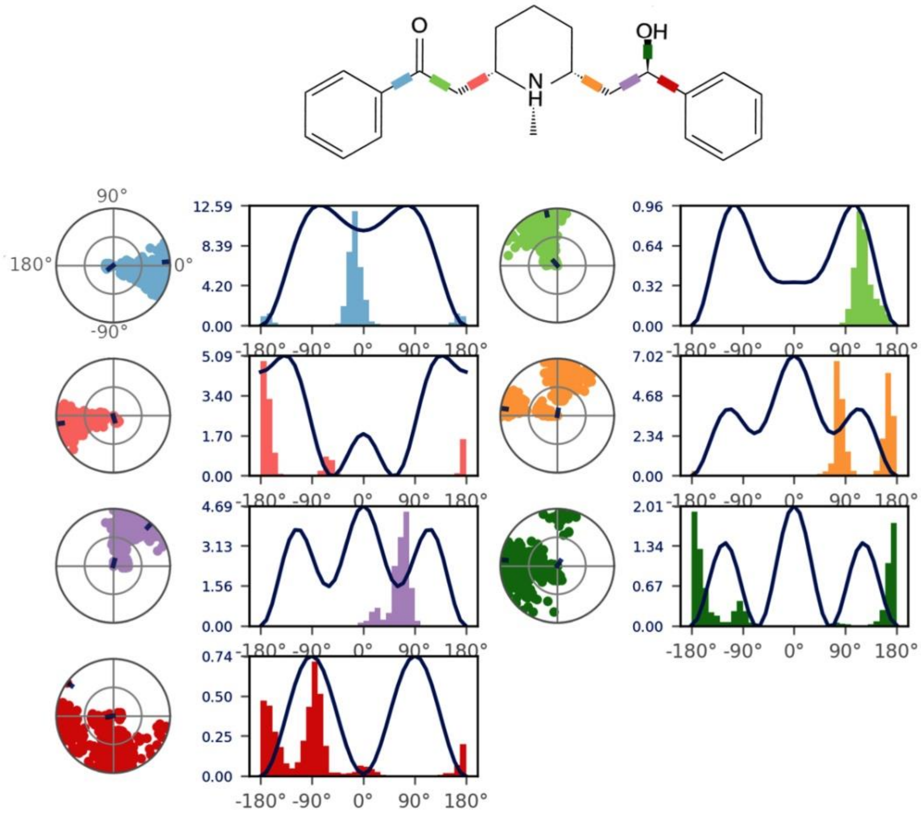

Figure S4: Torsion profile of the Lobeline during the simulations. The scattered radial plots indicate the more conformational freedom experienced by the rotatable bonds. All other details remain consistent with the information provided in Figure S3.

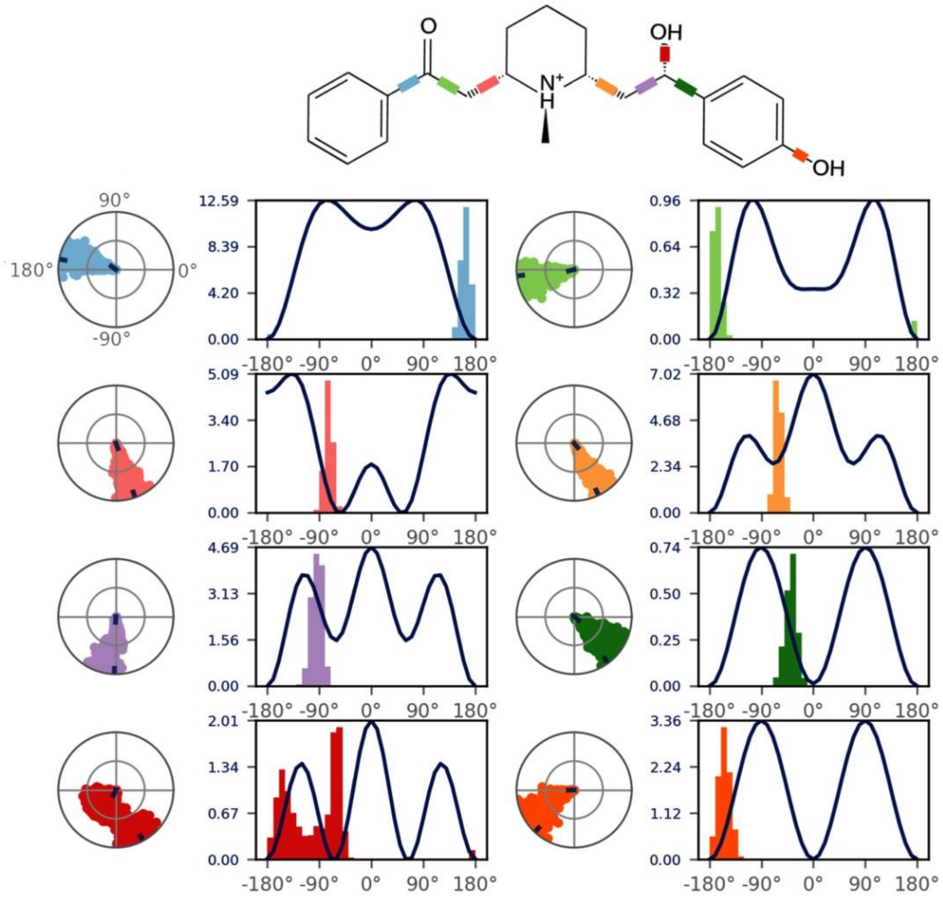

Figure S5: Torsion profile of the Lob-1 during the simulations. The scattered radial plots indicate the more conformational freedom experienced by the rotatable bonds. All other details remain consistent with the information provided in Figure S3.

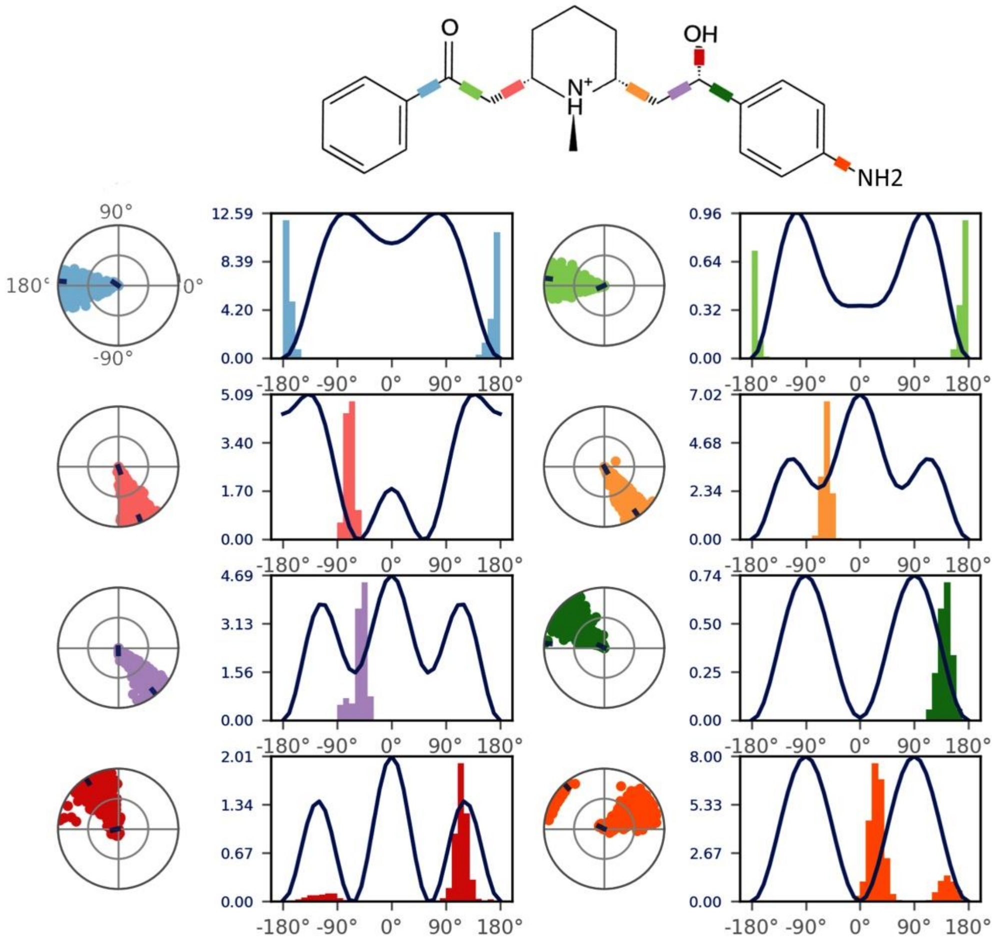

Figure S6: Torsion profile of the Lob-2 during the simulations. The scattered radial plots indicate the more conformational freedom experienced by the rotatable bonds. All other details remain consistent with the information provided in Figure S3.

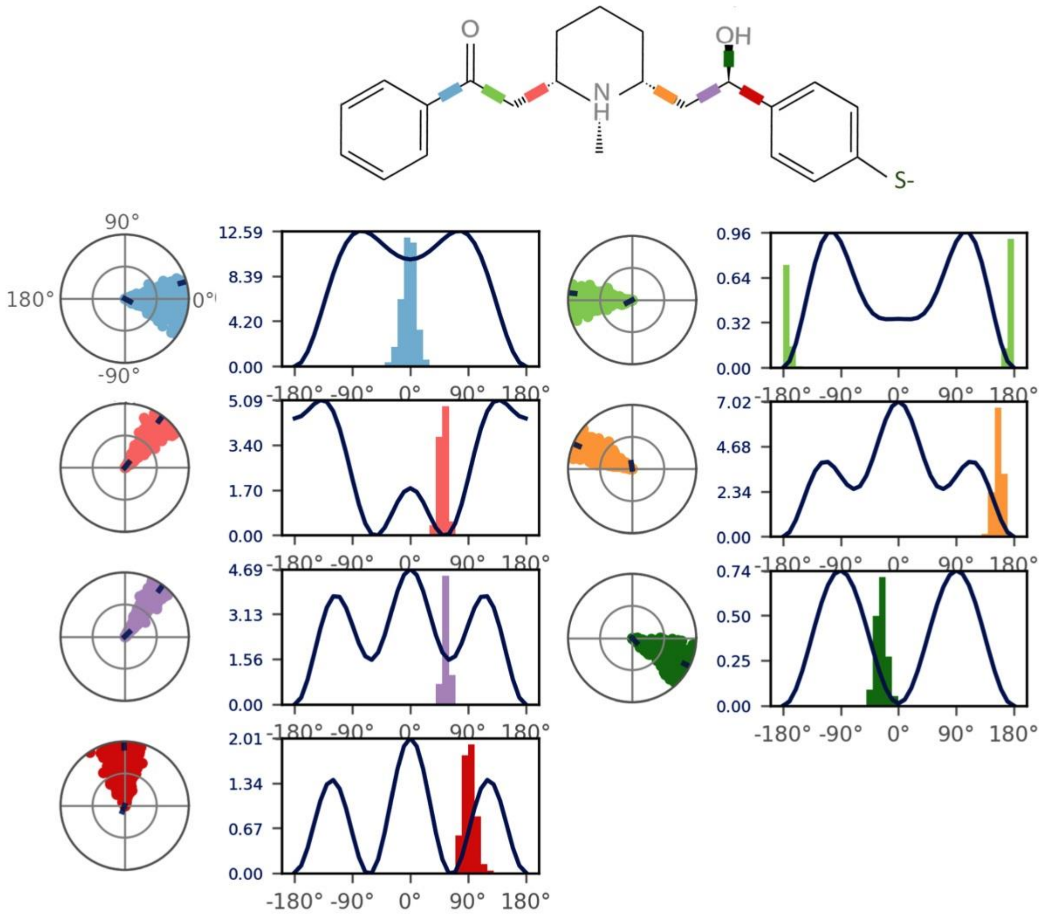

Figure S7: Torsion profile of the Lob-3 during the simulations. The scattered radial plots indicate the more conformational freedom experienced by the rotatable bonds. All other details remain consistent with the information provided in Figure S3.

Sl No	System	No. of atoms	Simulation time (ns)
1	NMDAR-Ifenprodil	70406	500
2	NMDAR-Lobeline	70404	500
3	NMDAR-Lob1	70327	500
4	NMDAR-Lob2	70331	500
5	NMDAR-Lob3	70309	500

Table S1: The number of atoms in the various NMDAR-ligand complexes along with simulation durations.
